# Etiology and prognosis of acute viral encephalitis and meningitis in Chinese children: a multicentre prospective study

**DOI:** 10.1186/s12879-017-2572-9

**Published:** 2017-07-14

**Authors:** Junhong Ai, Zhengde Xie, Gang Liu, Zongbo Chen, Yong Yang, Yuning Li, Jing Chen, Guo Zheng, Kunling Shen

**Affiliations:** 1 Key Laboratory of Major Diseases in Children, Ministry of Education, Beijing Key Laboratory of Pediatric Respiratory Infection Diseases, Virology Laboratory, Beijing Pediatric Research Institute, Beijing Children’s Hospital, Capital Medical University, National Center for Children’s Health, Beijing, 100045 China; 2 The Affiliated Hospital of Qingdao University, Shandong province, Qingdao, China; 3 The First Hospital of Yulin, Shanxi province, Yulin, China; 4 The First Hospital of Lanzhou University, Gansu province, Lanzhou, China; 5 Nanjing Children’s Hospital, Jiangsu province, Nanjing, China; 6 National Clinical Research Center for Respiratory Diseases, Key Laboratory of Major Diseases in Children, Ministry of Education, Respiratory Department, Beijing Children’s Hospital, Capital Medical University, National Center for Children’s Health, Beijing, 100045 China

**Keywords:** Viral encephalitis, Viral meningitis, Etiology, Prognosis, Children

## Abstract

**Background:**

In China, there were few studies about the pathogens of acute viral encephalitis and meningitis in children in recent years. The aims of this study were to characterize the etiology and prognosis of acute viral encephalitis and meningitis in Chinese children.

**Methods:**

This was a multicentre prospective study. Two hundred and sixty one viral encephalitis patients and 285 viral meningitis patients were enrolled. The mean age of viral encephalitis and meningitis were 5.88 ± 3.60 years and 6.39 ± 3.57 years, respectively. Real-time reverse transcription PCR and multiplex PCR were used to detect human enteroviruses and herpes viruses in cerebrospinal fluid (CSF) of patients with encephalitis or meningitis. The enzyme-linked immune absorbent assay (ELISA) was used for detecting IgM antibody against Japanese encephalitis virus (JEV) in CSF and against mumps virus, tick-borne encephalitis virus (TBEV), dengue virus and rubella virus in acute serum. The clinical and outcome data were collected during patients’ hospitalization.

**Results:**

The etiology of viral encephalitis was confirmed in 52.5% patients. The primary pathogen was human enteroviruses (27.7%) in viral encephalitis. The incidence of sequelae and the fatality rate of viral encephalitis with confirmed etiology were 7.5% and 0.8%, respectively. The etiology of viral meningitis was identified in 42.8% cases. The leading pathogen was also human enteroviruses (37.7%) in viral meningitis. The prognosis of viral meningitis was favorable with only 0.7% patients had neurological sequelae.

**Conclusions:**

Human enteroviruses were the leading cause both in acute viral encephalitis and viral meningitis in children. The incidence of sequelae and fatality rate of viral encephalitis with confirmed etiology were 7.5% and 0.8%, respectively. The prognosis of viral meningitis was favorable compared to viral encephalitis.

## Background

Encephalitis is a serious form of neurologic disease with inflammation of the brain parenchyma. More than 100 infectious, post-infectious, and immune-mediated conditions can cause encephalitis [1]. Viral encephalitis is the most common among them [2]. The etiology of viral encephalitis varies according to the geographical region. In the United States, Italy and Australia, the most commonly identified pathogen was herpes simplex virus in children and adults [2–4]. In Southern Vietnam, Japanese encephalitis virus (JEV) was the leading cause of viral encephalitis in children [5]. In Uttar Pradesh, India, enterovirus was an important cause of encephalitis in children [6]. However, herpes simplex virus was the most commonly identified pathogen in eastern India (children and adults) [7].

Meningitis is a disease that involves the membranes surrounding the central nervous system (CNS) and is characterized by fever, headache, nausea, vomiting, meningeal irritation, and alterations in the cerebrospinal fluid (CSF). Bacterial conjugate vaccines have dramatically changed the epidemiology of childhood meningitis and viral causes are increasingly predominant [8].

In China, there were few studies about the pathogens of acute viral encephalitis and meningitis in children in recent years. Two decades ago, Xu et al. reported that the most frequently identified pathogens of acute encephalitis in children were enteroviruses, followed by mumps and rubella [9]. However, there were limitations in their study such as small sample size and limited to Beijing only. What’s more, the epidemiology of viral encephalitis and meningitis may have changed by the use of vaccines. Thus, the aims of this study were to characterize the etiology and prognosis of acute viral encephalitis and meningitis in Chinese children.

## Methods

### Patients

This was a multicentre prospective study performed at five hospitals from June 2009 to October 2012 consecutively. The hospitals were Beijing Children’s Hospital, Capital Medical University, the Affiliated Hospital of Qingdao University, the First Hospital of Yulin, the First Hospital of Lanzhou University and Nanjing Children’s Hospital. These hospitals were located at Beijing, Shandong, Shanxi, Gansu and Jiangsu province, respectively. The patients come from the surrounding regions of each city. Thus, the study mainly represented the etiology of viral encephalitis and meningitis in northern China.

Inpatients suspected with viral encephalitis or meningitis were enrolled in the study on the first day of hospitalization.

The diagnostic criteria of acute viral encephalitis include: (1) acute onset; (2) altered mental status (defined as decreased or altered level of consciousness, lethargy or personality change) lasting ≥24 h with no alternative cause identified; (3) documented fever ≥38 °C (100.4 °F) within the 72 h before or after presentation; or generalized or partial seizures not fully attributable to a preexisting seizure disorder; (4) abnormality of brain parenchyma on neuroimaging suggestive of encephalitis; or abnormality on electroencephalography that is consistent with encephalitis; (5) no evidence of bacterial meningitis by microscopy and culture of CSF.

The diagnostic criteria of acute viral meningitis include: (1) acute onset of fever and symptoms such as headache, vomiting and/or nuchal rigidity; (2) absence of parenchymal involvement; (3) with no evidence of bacterial meningitis by microscopy and culture of CSF.

Patients with demyelinating, metabolic, toxic or neurological degenerative diseases and HIV infection were excluded.

### Clinical data and specimen collection

A form was designed for collecting clinical information from the medical records during hospitalization. The information included general demographic characteristics, clinical symptoms and physical signs, laboratory findings, neuroimaging and electroencephalography results and outcome of the patients. Information collection was completed by physician.

Acute phase CSF samples (0.4–1.0 ml) and acute serum samples (1.0–1.5 ml) were collected within 3 days after admission. All specimens were transported to the laboratory and stored at −80 °C before detection.

### Nucleic acid extraction from CSF

Viral nucleic acid was abstracted using the QIAamp MinElute Virus Spin Kit (QIAGEN, Germany, Cat. N. 57,704) from the CSF (0.2 ml), conducted by the manufacturer’s instruction. The elution volume was 50ul.

### Real-time reverse transcription polymerase chain reaction (RT-PCR) amplification assay for human enteroviruses

Human enteroviruses were screened using a real-time RT-PCR by enteroviruses universal nucleic acid detection kit (PCR - fluorescent probe) (DA AN GENE, China, Cat. N. DA-BT 109). The serotypes of enterovirus were not identified here.

### Multiplex PCR for herpes virus

For human enteroviruses negative samples, a multiplex PCR was tested for six herpes viruses which include herpes simplex virus 1 (HSV1), herpes simplex virus 2 (HSV2), varicella zoster virus (VZV), Epstein-Barr virus (EBV), cytomegalovirus (CMV) and human herpes virus 6 (HHV6) via the Seeplex® meningitis-V1 ACE Detection (V2.0) (Seegene, Korea, Cat. N. MG6611Y).

It has been reported that herpes virus DNA was detected in CSF of some patients with confirmed enterovirus meningitis [10]. However, the occurrence of coinfection of herpes viruses and enteroviruses in CNS seems to be a rather rare event in immunocompetent patients. What’s more, the reasons and clinical significance of coinfection of herpes viruses and enteroviruses are still unclear. In our pre-experiment, herpes virus DNA was not detected in CSF of patients with enterovirus encephalitis or meningitis. Therefore, we did not detect the herpes virus DNA in specimens with being positive of enterovirus.

### Enzyme-linked immune absorbent assay (ELISA)

The IgM antibody against JEV in CSF was detected by antibody capture ELISA. The IgM antibodies of mumps virus, tick-borne encephalitis virus (TBEV), Dengue virus and rubella virus in acute serum samples were detected by ELISA according to the manufacturer’s instructions (Anti-Mumps viruses ELISA (IgM) (EUROIMMUN, Germany, Cat. N. EI2630–9601 M); Anti-TBE virus ELISA (IgM) (EUROIMMUN, Germany, Cat. N. EI 2661–9601 M); Anti-Dengue Virus ELISA (IgM) (EUROIMMUN, Germany, Cat. N. EI 266b-9601 M) and Anti-Rubella Virus ELISA (IgM) (EUROIMMUN, Germany, Cat. N. EI 2590–9601 M), respectively.

### Etiology definition

The etiology of acute viral encephalitis and meningitis was defined if (1) the nucleic acid or specific IgM antibodies were positive in CSF; or (2) the specific IgM antibodies were positive in acute serum, and could not be explained with other diagnosis.

## Results

### Demographic characteristics of patients

Six hundred and forty two cases clinically diagnosed as viral encephalitis or viral meningitis were enrolled into this study from June 2009 to October 2012. Forty three patients were excluded according to the exclusion criteria and 53 cases were excluded for final diagnosis such as febrile convulsion, atypical purulent meningitis and so on.

**Fig. 1:**
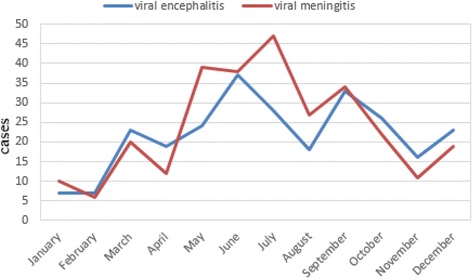
Seasonal distribution of viral encephalitis and meningitis cases

A total of 546 patients including 261 viral encephalitis patients with a mean age of 5.88 ± 3.60 years (range 0.17–16.0 years) and 285 viral meningitis patients with a mean age of 6.39 ± 3.57 years (range 0.42–14.22 years) were enrolled. The male to female ratio of viral encephalitis and meningitis was 1.97 and 2.24, respectively (Table 1). Paired CSF and acute serum samples were collected from 312 cases (169 viral encephalitis and 143 viral meningitis). Single CSF samples were obtained from other 234 (92 viral encephalitis and 142 viral meningitis) patients. The seasonal distribution of viral encephalitis and meningitis is shown in Fig. 1.

**Table 1:** Demographic characteristics of patients

Disease	Sex			Age (year)		
	Male	Female	Sex ratio	Minimum	Maximum	Mean
Encephalitis (*n* = 261)	173	88	1.97	0.17	16.00	5.88 ± 3.60
Meningitis (*n* = 285)	197	88	2.24	0.42	14.22	6.39 ± 3.57

### Definitive etiology of viral encephalitis and meningitis

**Fig. 2:**
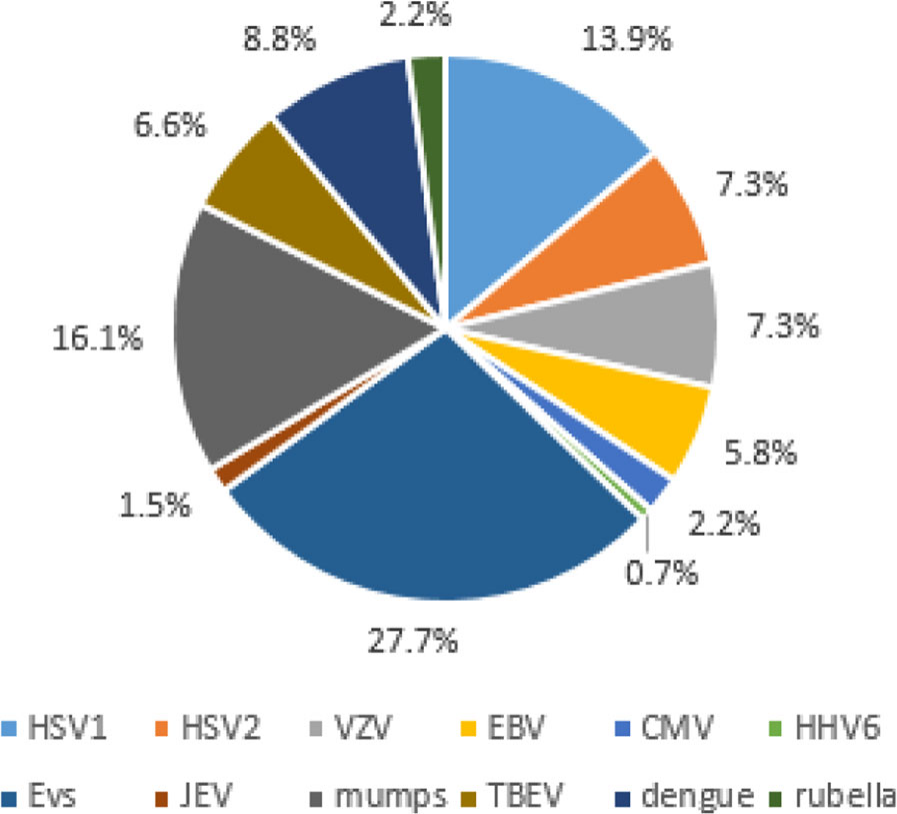
Percentage of viral pathogens in viral encephalitis cases with confirmed etiology. HSV1: herpes simplex virus 1; HSV2: herpes simplex virus 2; VZV: varicella zoster virus; EBV: Epstein-Barr virus; CMV: cytomegalovirus; HHV6: human herpes virus 6; EVs: human enteroviruses; JEV: Japanese encephalitis virus; mumps: mumps virus; TBEV: tick-borne encephalitis virus; dengue: Dengue virus; rubella: rubella virus

The etiology of acute viral encephalitis was confirmed in 52.5% (137/261) patients. The leading pathogen was human enteroviruses (27.7%, 38/137), followed by mumps virus (16.1%, 22/137) and HSV1 (13.9%, 19/137) (Fig. 2). JEV was rare in this study, detected only in 2 patients.

**Fig. 3:**
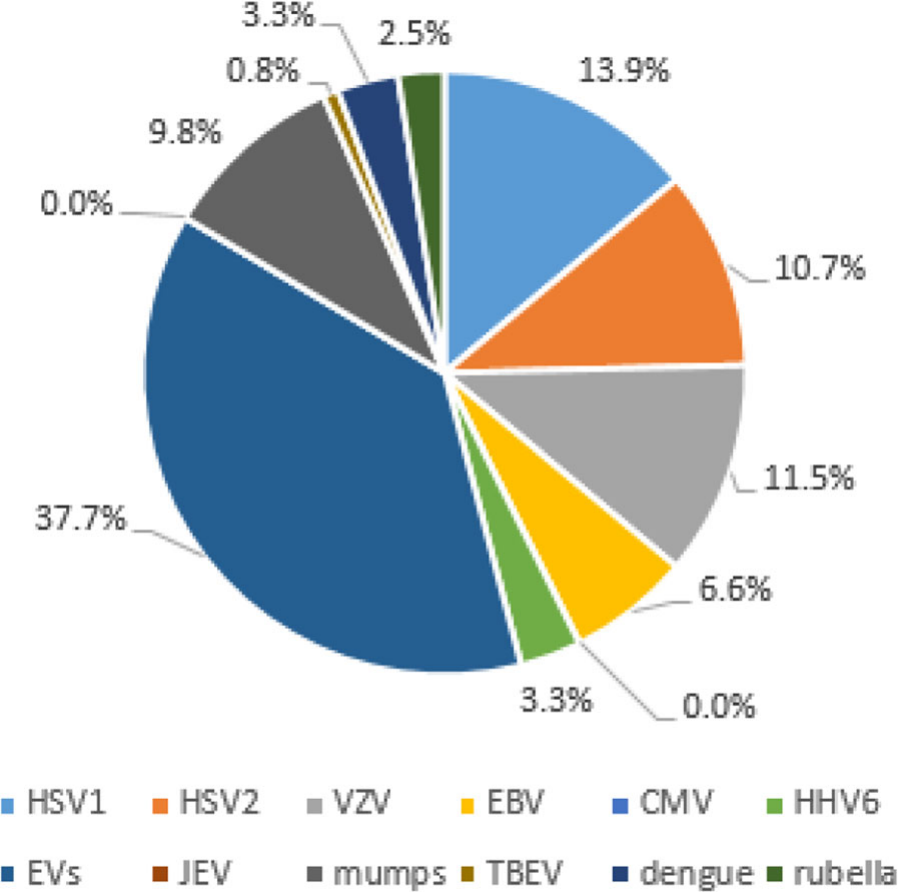
Percentage of viral pathogens in viral meningitis cases with confirmed etiology. HSV1: herpes simplex virus 1; HSV2: herpes simplex virus 2; VZV: varicella zoster virus; EBV: Epstein-Barr virus; CMV: cytomegalovirus; HHV6: human herpes virus 6; EVs: human enteroviruses; JEV: Japanese encephalitis virus; mumps: mumps virus; TBEV: tick-borne encephalitis virus; dengue: Dengue virus; rubella: rubella virus

The etiology of viral meningitis was identified in 42.8% (122/285) cases. The primary pathogen was also human enteroviruses (37.7%, 46/122), followed by HSV1 (13.9%, 17/122) and VZV (11.5%, 14/122) (Fig. 3). No JEV and CMV were detected.

### Seasonal distribution of viral encephalitis and meningitis cases with confirmed etiology

**Fig. 4:**
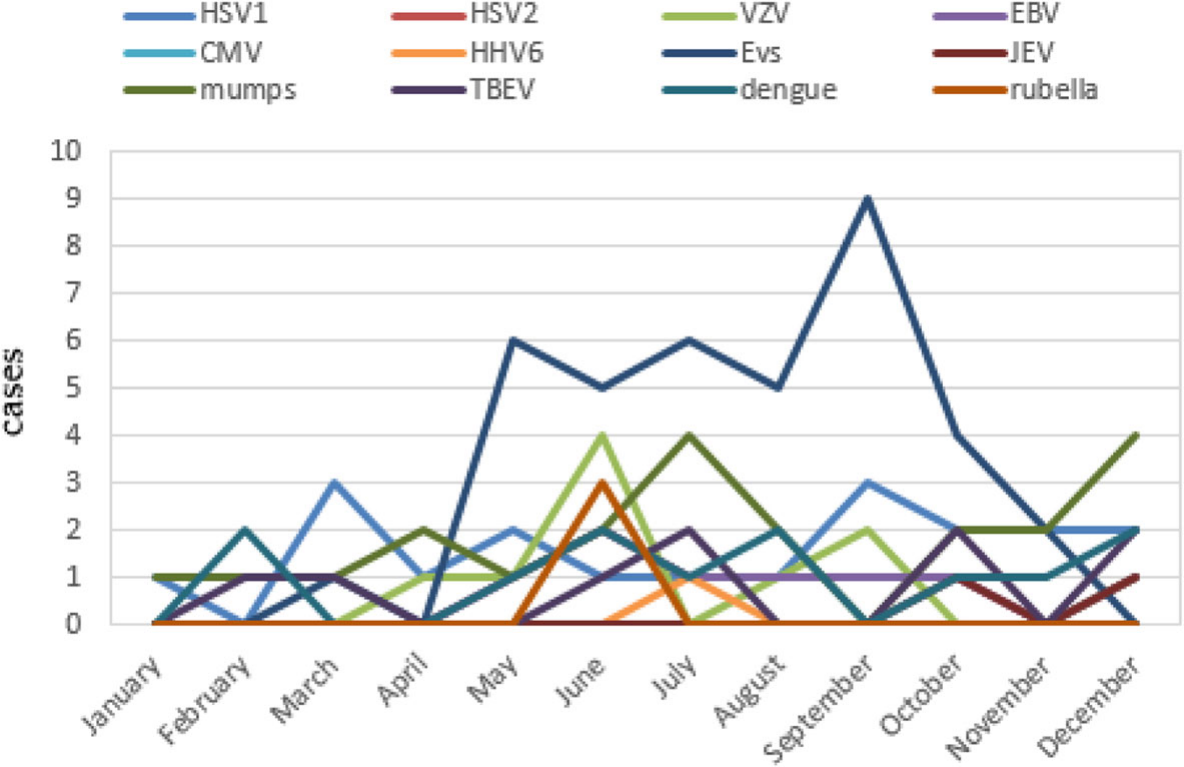
Seasonal distribution of viral encephalitis cases with confirmed etiology. HSV1: herpes simplex virus 1; HSV2: herpes simplex virus 2; VZV: varicella zoster virus; EBV: Epstein-Barr virus; CMV: cytomegalovirus; HHV6: human herpes virus 6; EVs: human enteroviruses; JEV: Japanese encephalitis virus; mumps: mumps virus; TBEV: tick-borne encephalitis virus; dengue: Dengue virus; rubella: rubella virus

**Fig. 5:**
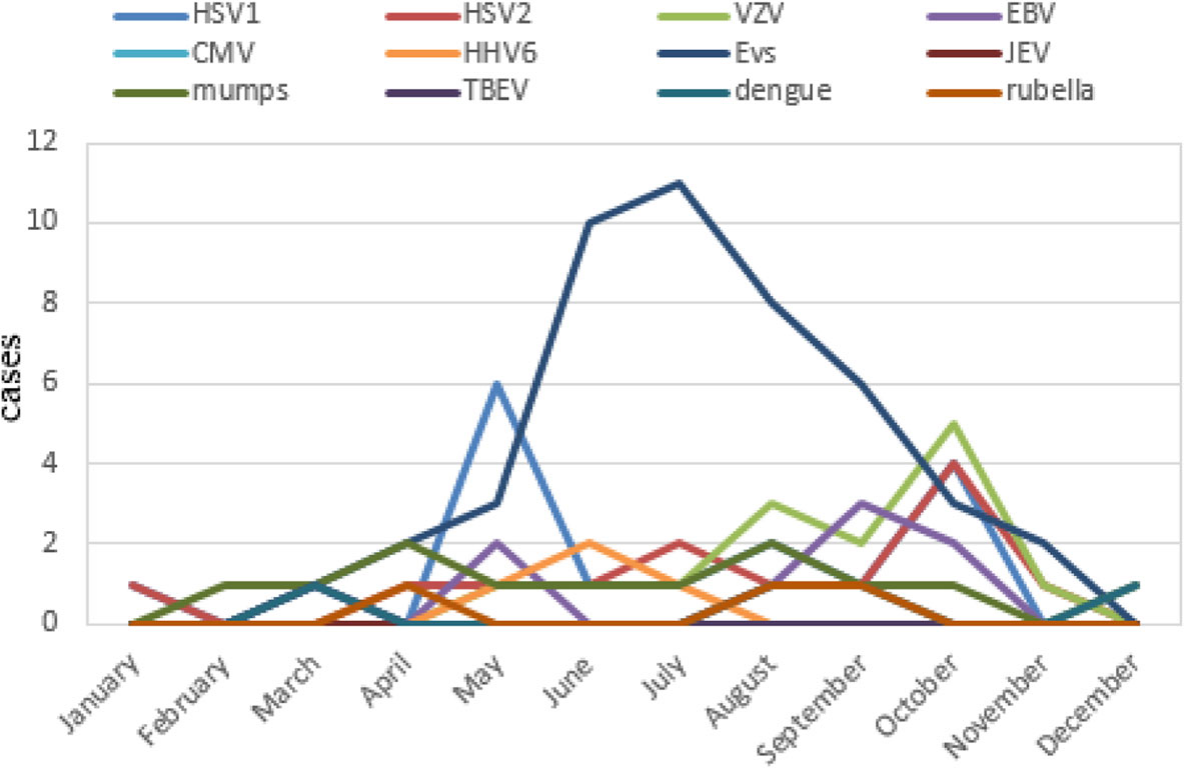
Seasonal distribution of viral meningitis cases with confirmed etiology. HSV1: herpes simplex virus 1; HSV2: herpes simplex virus 2; VZV: varicella zoster virus; EBV: Epstein-Barr virus; CMV: cytomegalovirus; HHV6: human herpes virus 6; EVs: human enteroviruses; JEV: Japanese encephalitis virus; mumps: mumps virus; TBEV: tick-borne encephalitis virus; dengue: Dengue virus; rubella: rubella virus

It is known that human enteroviruses infection is prevalent in summer and autumn. The seasonal distribution of human enterovirus encephalitis and meningitis were from May to October in this study, in accordance with the prevalence of the virus (shown in Figs. 4 and 5). There was no obvious seasonal trend in other viral encephalitis and meningitis.

### Prognosis of the viral encephalitis and meningitis patients

The outcome data was available from patients except 6 children with viral encephalitis who transferred to another hospital. All outcome data was collected at discharge. Patients of viral encephalitis and viral meningitis with neurological sequelae or died were shown in Table 2.

**Table 2:** Patients with neurological sequelae or death

Number	Diseases	Age(years)	Pathogens	Prognosis
1	encephalitis	1.4	HSV1	coma
2	encephalitis	6.5	HSV1	aphasia
3	encephalitis	4.0	HSV1	aphasia
4	encephalitis	5.0	VZV	coma
5	encephalitis	9.0	VZV	secondary epilepsy
6	encephalitis	1.0	VZV	cognitive impairment
7	encephalitis	3.9	VZV	blind
8	encephalitis	7.0	EBV	ataxia
8	encephalitis	6.0	CMV	dysphasia
10	encephalitis	2.2	EVs	death
11	encephalitis	1.0	JEV	hearing impaired
12	encephalitis	0.6	TBEV	hemiplegia
13	encephalitis	5.0	unknown	coma
14	encephalitis	7.0	unknown	death
15	encephalitis	4.0	unknown	visual impaired
16	encephalitis	4.0	unknown	euphoria
17	encephalitis	1.7	unknown	aphasia
18	encephalitis	10.3	unknown	secondary epilepsy
19	encephalitis	11.4	unknown	convulsion
20	encephalitis	6.8	unknown	hearing impaired
21	encephalitis	3.9	unknown	hemiplegia
22	meningitis	11.8	HSV1	headache
23	meningitis	11.8	unknown	speech difficulties

*HSV1* herpes simplex virus 1, *VZV* varicella zoster virus, *EBV* Epstein-Barr virus, *CMV* cytomegalovirus, *EVs* human enteroviruses, *JEV* Japanese encephalitis virus, *TBEV* tick-borne encephalitis virus

In viral encephalitis patients, 91.7% (234/255) got well, 7.5% (19/255) had neurological sequelae and 0.8% (2/255) died. The neurological sequelae included coma, aphasia, secondary epilepsy, cognitive impairment, blindness, ataxia, dysphasia, hearing impairment and hemiplegia. One patient died of unknown pathogen (Table 2). Another patient died of human enterovirus encephalitis. The fatality rate of human enterovirus encephalitis was 2.6% (1/38). No patients died of HSV1 and VZV encephalitis. In HSV1 and VZV encephalitis patients, 15.8% (3/19) and 40% (4/10) had neurological sequelae, respectively.

The prognosis of viral meningitis patients was favorable with 99.3% (283/285) recovery. There were only two (0.7%, 2/285) patients had neurological sequelae, one with unresolved headache (sporadically ongoing) and another with speech difficulties (Table 2).

## Discussion

This is a multicentre prospective study about the etiology and prognosis of viral encephalitis and meningitis in Chinese children. The results showed that the leading pathogen of viral encephalitis and meningitis were human enterovirus in Chinese children.

Human enteroviruses are the most common pathogen of viral meningitis in children worldwide [11]. Our results are consistent with data from other countries. But the most common pathogen of viral encephalitis varies in different countries and areas. HSV was the most common pathogen of viral encephalitis in France, England, the United States and eastern India [3, 7, 12–14]. However, in Southern Vietnam, the primary pathogen of viral encephalitis in children was JEV (26%) [5]. The most common pathogen of viral encephalitis in Chinese children was human enteroviruses in this study. This result is consistent with previous studies from China and Greece [15].

Japanese encephalitis (JE) is one of the most important viral encephalitis in the world. China was one of the most prevalent countries for JE in 1950s–1960s [16, 17]. Since JE vaccine became available in the late 1970s, the incidence of JE has declined significantly [18]. In 1980, China participated in the Expanded Program on Immunization (EPI) of WHO. In 2008, the JE vaccine was included in the national planned immunization. After that the number of JE cases in China has been decreased dramatically [19]. In this study, among 261 viral encephalitis patients, only 2 were JE patients. It is reported that JE is more prevalent in south China and the morbidity rates of JE in south and north China differed by 20-fold [20]. As for this multicentre study, the most cases came from northern China. Thus, the lower morbidity rate of JE in this study was consistent with other findings [20].

The fatality rate was 0.8% in this study, which is lower than a study conducted in southwest China where 2.5% patients died before being discharged from the hospital [21]. The different fatality rates may be contributed to the age difference of patients between the two studies. The mean age was 38.7 years (9–96) in the study of southwest China, while there were only children in our study.

The morbidity and mortality of viral encephalitis vary among different pathogens. Herpes simplex encephalitis (HSE) is the most common cause of sporadic viral encephalitis in Western countries. If untreated, the mortality rate associated with HSE is approximately 70% [22]. Despite antiviral therapy, the mortality is still higher than 30%, and almost 60% of surviving individuals develop neurological sequelae [22]. A recent international study suggested that rapid diagnosis and early administration of antiviral therapy at the onset of HSE are keys to a favorable outcome [23]. In this study, 15.8% (3/19) of HSV1 encephalitis patients had neurological sequelae and no patients died. This result is different from the data of Western countries. The rapid diagnosis, early supported and antiviral therapy may be the reason of favorable prognosis in this study.

This study has some limitations. First, there are more than 100 serotypes of human enteroviruses and the virulence varies in different serotypes [24]. The serotypes of human enteroviruses were not identified in this study. Second, it was not a long term follow-up study. The clinical data was collected during hospitalization.

## Conclusions

In conclusion, human enteroviruses were the most common etiology identified both in acute viral encephalitis and viral meningitis in children. The incidence of sequelae and fatality rate of viral encephalitis with confirmed etiology were 7.5% and 0.8%, respectively. The prognosis of viral meningitis was favorable compared to viral encephalitis.

## Abbreviations

CMV: Cytomegalovirus
CNS: Central nervous system
CSF: Cerebrospinal fluid
EBV: Epstein-Barr virus
ELISA: Enzyme-linked immune absorbent assay
HHV6: Human herpes virus 6
HSE: Herpes simplex encephalitis
HSV1: Herpes simplex virus 1
HSV2: Herpes simplex virus 2
JE: Japanese encephalitis
JEV: Japanese encephalitis virus
RT-PCR: Reverse transcription polymerase chain reaction
TBEV: Tick-borne encephalitis virus
VZV: Varicella zoster virus
